# Allergic rhinitis is a risk factor of gastro-esophageal reflux disease regardless of the presence of asthma

**DOI:** 10.1038/s41598-019-51661-4

**Published:** 2019-10-29

**Authors:** Yu-Min Kung, Pei-Yun Tsai, Yu-Han Chang, Yao-Kuang Wang, Meng-Shu Hsieh, Chih-Hsing Hung, Chao-Hung Kuo

**Affiliations:** 1 Department of Internal Medicine, Kaohsiung Municipal Hsiao-Kang Hospital, Kaohsiung, Taiwan; 2 Department of Nursing, Kaohsiung Municipal Hsiao-Kang Hospital, Kaohsiung, Taiwan; 3 Teaching and research center, Kaohsiung Municipal Hsiao-Kang Hospital, Kaohsiung, Taiwan; 4 Division of Gastroenterology, Department of Internal Medicine, Kaohsiung Medical University Hospital, Kaohsiung, Taiwan; 5 Department of Medicine, Faculty of Medicine, College of Medicine, Kaohsiung Medical University, Kaohsiung, Taiwan; 6 Department of Pediatrics, Kaohsiung Medical University Hospital, Kaohsiung, Taiwan; 7 Department of Pediatrics, Faculty of Pediatrics, College of Medicine, Kaohsiung Medical University, Kaohsiung, Taiwan; 8 Research Center for Environmental Medicine, Kaohsiung Medical University, Kaohsiung, Taiwan

**Keywords:** Gastroenterology, Risk factors

## Abstract

Gastroesophageal reflux disease (GERD) can cause several upper airway symptoms and alter the physiology of nasopharyngeal mucosa, while upper airway diseases in turn might also exacerbate GERD symptoms. For a long time, asthma was considered a risk factor of GERD in the literature. Asthma and allergic rhinitis (AR) are usually identified as united airway disease according to similar epidemiology and pathophysiology; however, the association between AR and GERD is less elucidated. We aimed to evaluate whether AR would increase the development of GERD. Patients diagnosed as AR were identified from the National Health Insurance Research Database between January 1, 2000 and December 31, 2005 without prior history of gastroesophageal reflux disease. The outcome of interest was new-onset GERD. Cox regression models were applied to calculate the hazard ratio (HR) of GERD. We analyzed the data of 193,810 AR patients aged 18 years or older and being free of AR at baseline. The AR cohort (n = 96,905) had a significantly increased risk of GERD over a non-AR cohort (n = 96905) (adjusted HR (aHR) 1.94; 95% CI = 1.88–1.99, p < 0.001). AR may have stronger correlation with GERD than does asthma, although asthma might increase GERD risk by means of certain pathways shared with AR.

## Introduction

Gastro-esophageal reflux disease (GERD) is one of the most prevalent gastrointestinal diseases and its prevalence is increasing worldwide^1^. It is defined by its troublesome symptoms and/or complications caused by the refluxed contents from the stomach, creating a great adverse impact on health and quality of life as well as consuming financial resources in medical insurance^2^. The typical symptoms of GERD are regurgitation and heartburn. In addition to cardinal symptoms, reflux from stomach also induces so-called atypical or extra-esophageal symptoms, including cough, hoarseness, pharyngitis, laryngitis, chest pain, sinusitis, otitis media, dental erosions and sleep disturbance. Since there are many factors contributing to the development of reflux and reflux-related symptoms, GERD is identified as a multi-factorial disease. Even today, the pathophysiology of GERD is not fully understood and many mechanisms have been proposed to explain the development of GERD, inclusive of obesity, transient lower-esophageal sphincter relaxations (TLESRs), hiatus hernia, acid pockets, visceral hypersensitivity, impaired esophageal mucosal integrity, poor esophageal clearance, and delayed gastric emptying^3–5^.

In the literature and clinical practice, certain diseases are observed to be associated with GERD. Asthma is the most studied disease in its relationship with GERD^6^. Asthma is a complex condition characterized by variable and recurrent symptoms as the result of reversible airflow obstruction, bronchial hyper-responsiveness, and chronic airway inflammation. In a meta-analysis, asthma prevalence is 4.6% in patients with GERD and 3.9% in controls, while GERD symptom prevalence is 59.2% in patients with asthma and 38.1% in controls. Odds ratio is 2.26 for asthma in GERD, while it is 5.45 for GERD in asthma^7^. Cough and increased respiratory effort might induce reflux in patients with asthma^8^. On the other hand, exposure to acid content reflux may exacerbate asthma as a result of direct pulmonary tree damage as well as bronchoconstriction indirectly via vagal stimulation^9^. Asthma and allergic rhinitis (AR) are common airway diseases and are viewed as a united airway disease. They frequently occur together and share similar physiological traits, including heightened reactivity to a variety of stimuli and heightened bronchial hyper-responsiveness^10^.

AR is defined as an inflammatory process in the nasal mucosa that is stimulated by exposure to allergens. AR is very common, affecting 9 to 42% of children and 10 to 30% of adults^11,12^. Sneezing, nasal congestion, nose/throat itching, and anterior/posterior rhinorrhea are the four cardinal symptoms of AR. The immunopathology of AR and asthma are quite similar in terms of cellular influx of mast cells, eosinophils, and T-helper type 2(Th2) cells. In addition, similar mediators can also be found in patients with AR and asthma, such as histamine, cysteinyl leukotrienes, chemokines, and Th2 cytokines^10^.

As above, there is evidence of an association between asthma and GERD; however, few studies have evaluated the impact of AR on GERD. The aim of this study, which used the Taiwan National Health Insurance Research Database (NHIRD) database, was to access the relationship of AR with newly developed GERD in adults. However, we used ICD-9 codes of diagnosis for patient selection, as it is difficult to validate the accuracy of the diagnosis set by the treating doctor.

## Results

**Figure 1:**
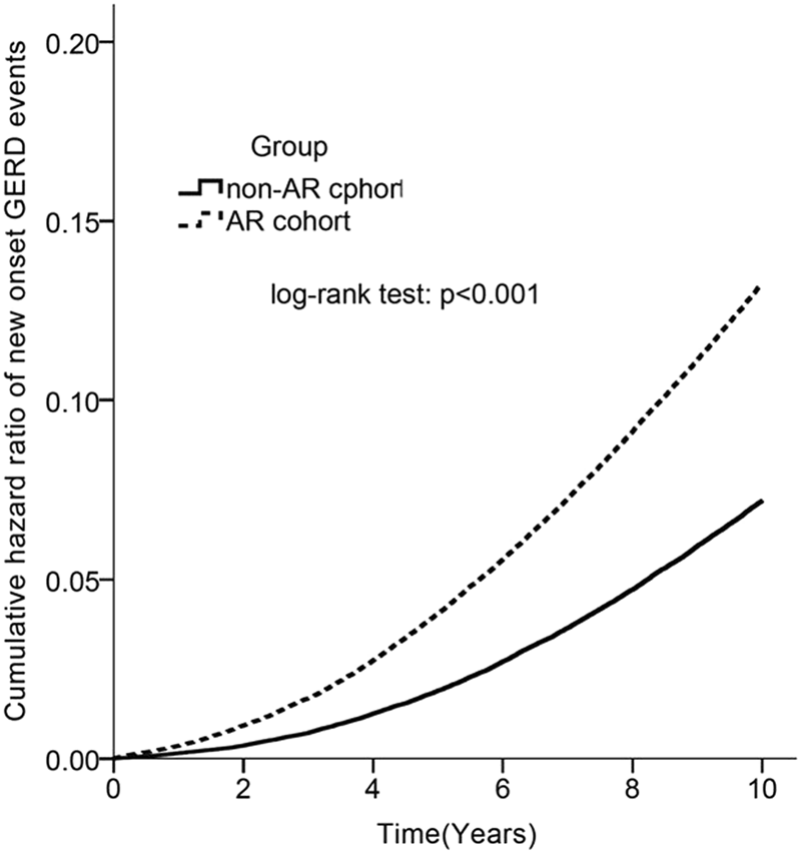
Kaplan–Meier curves used to show overall cumulative hazard rate of new onset GERD events between non-AR cohort and AR cohort followed by time (years). The hazard ratio is greater in the AR cohort than in the non-AR cohort (p < 0.001).

From Jan 1, 2000 to Dec 31, 2005, we conducted a study with 96,905 and 96,905 children as AR cohort and non-AR cohort controls respectively from LHID2005. The mean age of the AR cohort was 43.72 ± 16.9 years. The distribution of age, gender, and comorbidities were similar between AR cohort and non-AR cohort groups (Table 1). The overall cumulative hazard rate of new onset GERD events followed by time was greater in the AR cohort than in the non-AR cohort (p < 0.001) (Fig. 1).

**Table 1:** Demographic data between non-AR and AR cohorts (N = 193810).

	non-AR cohort (n = 96905)		AR cohort (n = 96905)		p value
	n	(%)	n	(%)	
Age, (mean, SD)	43.72	(16.9)	43.94	(16.9)	
<40	47396	(48.9)	47550	(49.1)	0.108
40–59	31169	(32.2)	31403	(32.4)	
>=60	18340	(18.9)	17952	(18.5)	
Gender					
Female	53470	(55.2)	53229	(54.9)	0.271
Male	43435	(44.8)	43676	(45.1)	
Comorbidities					
Diabetes	8667	(8.9)	8429	(8.7)	0.057
Hyperlipidemia	14189	(14.6)	13522	(14.0)	<0.001
Hypertension	20255	(20.9)	19550	(20.2)	<0.001
Atopic Dermatitis	2868	(3.0)	2933	(3.0)	0.394
Chronic sinusitis	27904	(28.8)	28373	(29.3)	0.019
Rhinitis	4580	(4.7)	4622	(4.8)	0.654
Nasal polyps	315	(0.3)	289	(0.3)	0.289
Anaphylaxis	22	(0.0)	22	(0.0)	>0.999
Depression	4061	(4.2)	4050	(4.2)	0.901

**Figure 2:**
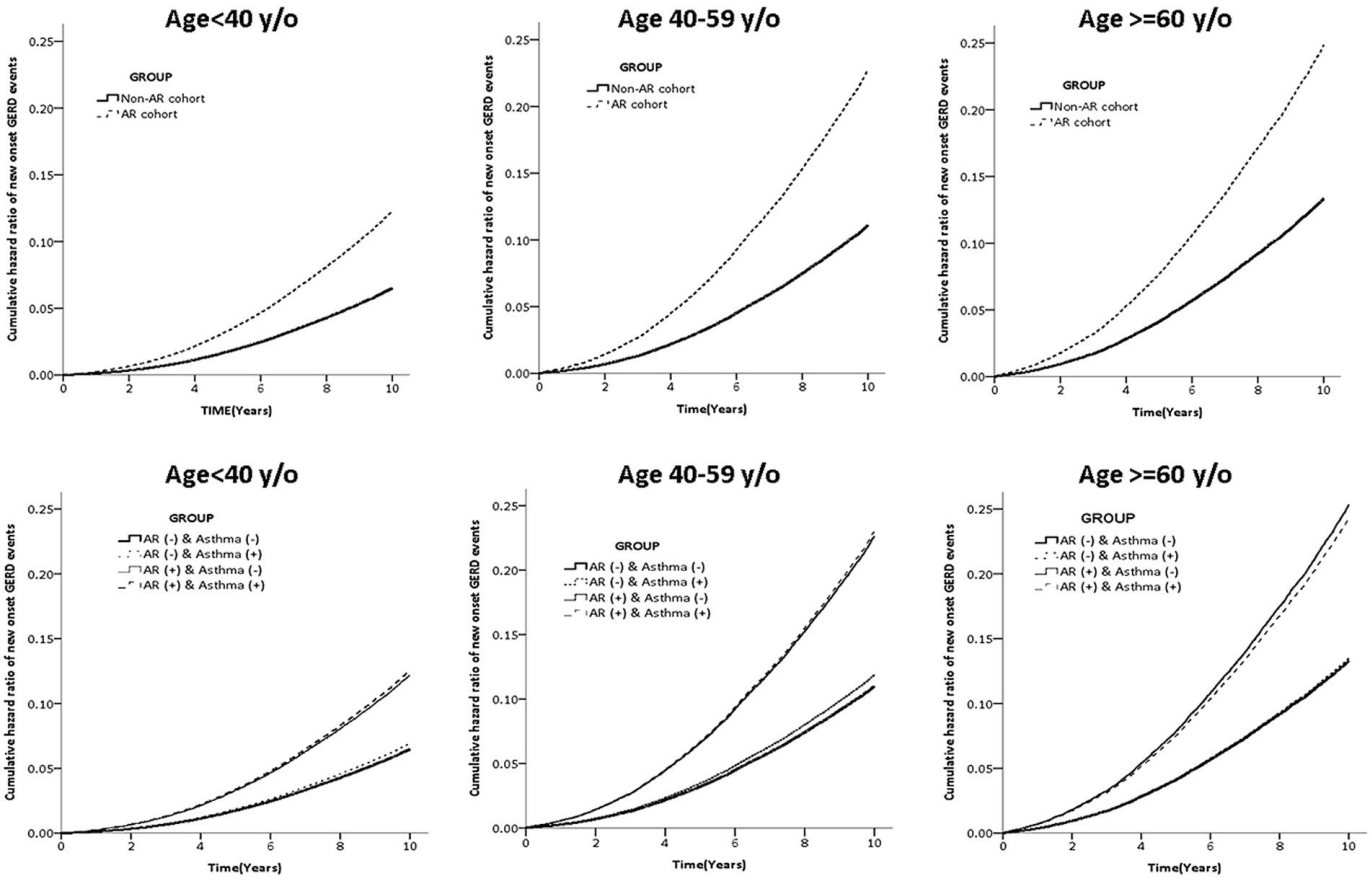
Kaplan–Meier curves used to estimate the probability of GERD stratified by different age stratification (Age < 40 y/o, 40–59 y/o, and ≧60 y/o). The hazard ratios are all higher in the AR cohort than in the non-AR cohort by different age stratification. We also divided non-AR and AR cohorts to Asthma (+) or Asthma (−). Asthma seemed less effective on cumulative hazard ratio of new-onset GERD events than was AR.

Kaplan–Meier curves were used to estimate the probability of GERD stratified by different age stratification (Fig. 2). The AR cohort and non-AR cohort groups followed an average of 8.71 years, and 8.86 years, while 14,365(14.8%) and 7,698(7.94%) of the two cohorts respectively developed new onset GERD within 10 years. Cox proportional hazard regression analysis revealed that the AR cohort was 94% more likely to develop GERD than the non-AR cohort (adjusted hazard ratio (aHR), 1.94; 95% CI = 1.88–1.99; p < 0.001). The increased risk of GERD remained significant after adjusting for potential co-variables in different age stratification levels (<40 years: adjusted HR, 1.88; 95% CI = 1.80–1.97; p < 0.001, 40–59 years: adjusted HR, 2.04; 95% CI = 1.96–2.13, p < 0.001; >=40 years: adjusted HR, 1.86; 95% CI = 1.75–1.97, p < 0.001) (Table 2).

**Table 2:** The risk of GERD between non-AR and AR cohorts (N = 193810).

	Case no.	per 1000 person year	Adjusted hazard ratio	(95% CI)	p value
Overall					
non-AR cohort	7698	9.0	Ref.		
AR cohort	14365	17.0	1.94	(1.88–1.99)	p < 0.001
Age, years					
<40 years	(N = 94946)				
non-AR cohort	2904	6.6	Ref.		
AR cohort	5267	12.1	1.88	(1.80–1.97)	p < 0.001
40–59 years	(N = 62572)				
non-AR cohort	3100	11.0	Ref.		
AR cohort	6027	22.0	2.04	(1.96–2.13)	p < 0.001
>=60 years	(N = 36292)				
non-AR cohort	1694	12.4	Ref.		
AR cohort	3071	22.8	1.86	(1.75–1.97)	p < 0.001

Adjusted for age, gender, comorbidities.

After adjusting for age, gender, and comorbidities, there were associated risk factors with increased risk of GERD, followed by age (<40 years as reference group, 40–59 years: adjusted HR 1.72, 95% CI 1.65–1.79, p < 0.001; >=60 years: adjusted HR 1.75, 95% CI 1.66–1.84, p < 0.001), gender (female as reference group, adjusted HR 0.93, 95% CI 0.90–0.96, p < 0.001), diabetes(adjusted HR 1.09, 95% CI 1.03–1.15, p < 0.001), hyperlipidemia (adjusted HR 1.45, 95% CI 1.38–1.52, p < 0.001), atopic dermatitis (adjusted HR 1.10, 95% CI 1.05–1.15, p < 0.001), chronic sinusitis (adjusted HR 1.37, 95% CI 1.32–1.42, p < 0.001), rhinitis (adjusted HR 1.33, 95% CI 1.24–1.42, p < 0.001), and depression (adjusted HR 1.17, 95% CI 1.08–1.27, p < 0.001) (Table 3).

**Table 3:** The risk factors of GERD in AR patients (N = 193810).

	Case no.	(%)	Adjusted hazard ratio	(95% CI)	p value
Age					
<40	5267	(11.1)	ref.		
40–59	6027	(19.2)	1.72	(1.65–1.79)	<0.001
>=60	3071	(17.1)	1.75	(1.66–1.84)	<0.001
Gender					
Female	8178	(15.4)	ref.		
Male	6187	(14.2)	0.93	(0.90–0.96)	<0.001
Comorbidities					
Diabetes					
no	12706	(14.4)	ref.		
yes	1659	(19.7)	1.09	(1.03–1.15)	0.002
Hyperlipidemia					
no	11348	(13.6)	ref.		
yes	3017	(22.3)	1.45	(1.38–1.52)	<0.001
Hypertension					
no	10729	(13.9)	ref.		
yes	3636	(18.6)	1.10	(1.05–1.15)	<0.001
Atopic Dermatitis					
no	13861	(14.8)	ref.		
yes	504	(17.2)	1.23	(1.13–1.35)	<0.001
Chronic sinusitis					
no	9407	(13.7)	ref.		
yes	4958	(17.5)	1.37	(1.32–1.42)	<0.001
Rhinitis					
no	13473	(14.6)	ref.		
yes	892	(19.3)	1.33	(1.24–1.42)	<0.001
Nasal polyps					
no	14321	(14.8)	ref.		
yes	44	(15.2)	0.91	(0.68–1.23)	0.560
Anaphylaxis					
no	14363	(14.8)	ref.		
yes	2	(9.1)	0.48	(0.12–1.91)	0.299
Depression					
no	13736	(14.8)	ref.		
yes	629	(15.5)	1.17	(1.08–1.27)	<0.001

Adjusted for age, gender, comorbidities.

Table 4 shows the additive effects of allergic rhinitis and asthma. Allergic rhinitis with asthma was associated with higher risk for new onset GERD more than the reference group (AR (−) & asthma (−)) (adjusted HR 1.98, 95% CI 1.90–2.05, p < 0.001). The increased risk of GERD remained significant after adjusting for potential co-variables in different age stratification levels (<40 years: adjusted HR, 1.93; 95% CI = 1.80–2.07; p < 0.001, 40–59 years: adjusted HR, 2.11; 95% CI = 1.99–2.24, p < 0.001; >=40 years: adjusted HR, 1.82; 95% CI = 1.68–1.96, p < 0.001).

**Table 4:** The additive effect of asthma on GERD between AR and non-AR cohorts (N = 193810).

		Case no.	per 1000 person year	Adjusted hazard ratio	(95% CI)	p value
Overall						
AR (−) & Asthma (−)	(n = 86258)	6700	8.7	ref.		
AR (−) & Asthma (+)	(n = 10647)	998	11.2	1.06	(0.99–1.13)	0.069
AR (+) & Asthma (−)	(n = 69299)	9879	16.3	1.94	(1.88–2.00)	<0.001
AR (+) & Asthma (+)	(n = 27606)	4486	18.9	1.98	(1.90–2.05)	<0.001
<40 years of age						
AR (−) & Asthma (−)	(n = 44493)	2699	6.5	ref.		
AR (−) & Asthma (+)	(n = 2903)	205	7.5	1.07	(0.93–1.24)	0.313
AR (+) & Asthma (−)	(n = 37518)	4081	11.9	1.85	(1.77–1.95)	<0.001
AR (+) & Asthma (+)	(n = 10032)	1186	12.8	1.93	(1.80–2.07)	<0.001
40–59 years of age						
AR (−) & Asthma (−)	(n = 27886)	2719	10.8	ref.		
AR (−) & Asthma (+)	(n = 3283)	381	12.9	1.09	(0.98–1.22)	0.094
AR (+) & Asthma (−)	(n = 22016)	4107	21.4	2.05	(1.95–2.15)	<0.001
AR (+) & Asthma (+)	(n = 9387)	1920	23.2	2.11	(1.99–2.24)	<0.001
>=60 years of age						
AR (−) & Asthma (−)	(n = 13879)	1282	12.3	ref.		
AR (−) & Asthma (+)	(n = 4461)	412	12.8	1.01	(0.90–1.13)	0.839
AR (+) & Asthma (−)	(n= 9765)	1691	23.1	1.90	(1.76–2.04)	<0.001
AR (+) & Asthma (+)	(n = 8187)	1380	22.4	1.82	(1.68–1.96)	<0.001

Adjusted for age, gender, comorbidities.

Table 5 reveals the average duration of new onset GERD between non-AR and AR cohorts. The overall duration in the non-AR cohort was 6.22 ± 2.43 years, while duration in the AR cohort was 5.93 ± 2.52 years, with a p value of <0.001.

**Table 5:** The average duration of new onset GERD between non-AR and AR cohorts.

	non-AR cohort		AR cohort		p value
	Mean	(SD)	Mean	(SD)	
Overall age	6.22	(2.43)	5.93	(2.52)	<0.001
<40 years	6.44	(2.34)	6.15	(2.46)	<0.001
40–59 years	6.22	(2.41)	5.92	(2.53)	<0.001
>=65 years	5.84	(2.57)	5.56	(2.55)	<0.001

## Discussions

Our results indicate that AR is significantly associated with the development of newly diagnosed GERD in adults. The duration of new onset of GERD development in the AR group was also shorter than in the non-AR group (Table 5). In addition, our comparison of the interaction of AR and asthma on GERD revealed that only the positive AR group as well as the coexistence of AR and asthma group increased the risk of GERD development (Table 4). The effect on GERD is stronger in the AR (+) & Asthma (+) group than in the AR (+) & Asthma (−) group, especially for subjects with age between 40 and 50 years. Exceptionally, the effect of the AR (+) & Asthma (+) group diminished in the group above 60 years of age. The same situation was observed in the risk of GERD between non-AR and AR cohorts (Table 2). One explanation may be that immunity response declines at ages above 60 years.

Asthma and AR are very common atopic conditions and chronic airway diseases. The prevalence of asthma ranges between 5 and 16%^13^. AR affects 5~22% of people worldwide^14^. Although about 19~38% of patients with AR have concomitance with asthma, there is a much higher frequency of patients with asthma having concomitant AR^15^. In a Finnish twin cohort from 1975 to 1990, there was a threefold risk of developing asthma with a diagnosis of AR and AR is typically diagnosed before asthma, indicating AR as a risk for development of new asthma^16,17^. As a result, AR is a common risk factor of newly onset asthma and GERD.

In patients with AR, increased frequency of swallowing is usually observed due to throat itching and posterior nasal dripping. The frequent swallowing would exacerbate the reflux by means of increased TLESRs. AR and its effect on nasal mucosa could give rise to similar effects on laryngeal mucosa including congestion, edema and excessive mucous secretion, which leads to symptoms of laryngopharyngeal reflux (LPR), a subgroup of GERD^18^. GERD can also be caused by the release of histamine from mast cells, which might promote the onset of this disease through lower-esophageal sphincter contractions^19^. Eosinophils, a key component of the allergic inflammation, are also discovered in esophageal mucosa of GERD patients^20^. No previous studies have examined the simultaneous presence of AR and asthma on the development of GERD. Asthma may predispose an individual to GERD by a variety of mechanisms, such as vagus nerve dysfunction, increased intrathoracic pressure, and altered diaphragmatic crural function^21,22^. Reflux may give rise to asthma either by the effects on the airway through an aspiration-induced response directly or by neurogenically-induced inflammation indirectly^23^. The causal correlation between asthma and GERD is hard to establish since either condition is able to induce the other. However, in our studies, the results demonstrated AR increased the risk of new-onset GERD instead of asthma without concomitance of AR. This might imply AR plays a more important role in inducing GERD than does asthma.

In our study, we also evaluated the risk factors of GERD in AR patients (Table 3). Older age, the female gender, and some comorbidities including diabetes, hyperlipidemia, hypertension, atopic dermatitis, chronic sinusitis, rhinitis and depression were identified as risk factors of GERD. This result is similar to that in previous literature. On the other hand, nasal polyps and anaphylaxis had no statistical significance on development of GERD. Patients with chronic rhinosinusitis with nasal polyps and simultaneous bronchial asthma suffered from significant extraesophageal reflux^24^. However, the role of allergy in chronic rhinosinusitis with and without nasal polyps has remained controversial^25^. Anaphylaxis is a serious allergic reaction that is rapid in onset and typically presents many different symptoms over minutes and hours. The most common symptoms included an itching rash, throat or tongue swelling, vomiting, shortness of breath and low blood pressure. It is caused by the release of inflammatory mediators and cytokines from mast cells and basophils, typically due to an immunological reaction^26^. Since anaphylaxis is a short-term immunity response, it has less impact on the chronic development of GERD. Additionally, there was too small a sample size of anaphylaxis incidences in our cohort.

Our studies had several limitations in that we did not consider confounding by lifestyle, obesity, smoking and alcohol consumption. Moreover, the diagnosis of AR and GERD was according to ICD-9 code. The tools for exact diagnosis of AR and GERD might be dissimilar, thus under-diagnosis or over-diagnosis might lead to inaccurate interpretation. Besides, the evaluation of the associated risks and diseases was also according to ICD-9 code, and the misclassification of ICD-9 could lead both to under- and overestimates of the diseases and the risks. Using a register-based data on clinical visits for allergic rhinitis and GERD means that a large number of subjects in the population suffering from allergic rhinitis and/or GERD would not be included in the study. Some patients with milder symptoms might self-medicate instead of seeking medical care. It is therefore likely that our study includes the more severe cases of allergic rhinitis and GERD. On the other hand, the peak onset of AR is around 20 years of age. In our study, the mean diagnosis age of AR was 44 years, but not the onset age; the reason might be that many younger patients prefer to self-medicate; thus, the exact period of diagnosis of GERD after onset of AR might well be longer.

In conclusion, our results provide evidence that AR, especially with asthma, has an increased risk factor for developing new-onset GERD in adults. Asthma might increase the risk of GERD by means of certain mechanisms shared with AR; however, the precise interaction and causal relationship between AR, asthma, and GERD is worth further investigation.

## Materials and Methods

### Data source

The study database used claims data from the National Health Insurance Research Database (NHIRD), which is managed by the Taiwan National Health Research Institute (NHRI). The NHIRD contains comprehensive healthcare information for virtually all of Taiwan’s nationally insured population, including demographic data, clinic visit date, diagnostic codes, and prescription details. For the study, we used a subset of NHIRD known as the Longitudinal Health Insurance Database (LHID) 2005, which contains ambulatory and inpatient medical care records for one million randomly sampled subjects enrolled into the NHI system in the year of 2005. According to the NHRI, there were no statistically significant differences in age, gender, or healthcare costs between the LHID 2005 and NHIRD.

### Study patients

We identified allergic rhinitis (ICD-9 code: 477.8, 477.9) between January 1, 2000 and December 31, 2005 for the AR cohort. The AR cohort group was compared with a comparison cohort, which consisted of patients who had never been diagnosed as having allergic rhinitis, matched (1:1) on the basis of age, sex, comorbidities, propensity score and index year. The date of first diagnosis of allergic rhinitis for each patient was defined as the index date. Subjects were excluded if they were less than 18 years of age, had gastroesophageal reflux disease (GERD) diagnosed before the index date, or had incomplete follow-up insurance data. The protocol was approved by the Institutional Review Board of Kaohsiung Medical University Hospital (KMUHIRB-F(I)-20150168). All methods were performed in accordance with the relevant guidelines and regulations.

### Diagnosis of gastroesophageal reflux disease (GERD)

The outcome of interest was a diagnosis of ICD-9-CM 530.11 (gastroesophageal reflux disease (GERD)). Patients were followed from the index date to the new onset of gastroesophageal reflux disease, death, disenrollment from the national health insurance, or the end of the study date (December 31, 2013), whichever came first.

### Potential confounders

Inpatient and outpatient files from the year prior to the index date were used to obtain information on co-morbidities including atopic dermatitis (ICD-9 code: 691.8), chronic sinusitis (ICD-9 code: 472.0), rhinitis (ICD-9 code: 461,473), and nasal polyps (ICD-9 code: 471) were evaluated.

### Statistics analysis

Pearson chi-square test or Fisher’s exact test was used to evaluate differences in categorical variables between AR and non-AR cohorts. Independent *t*-test evaluated differences of two cohorts in continuous variables. Cox proportional-hazards regression analysis was performed to examine the risk of GERD in the AR cohort compared with the non-AR cohort during the follow-up period. Several co-variables, including age, gender, and co-morbidities, were adopted in the statistical analysis model. Hazard ratios (HR) and 95% confidence intervals (CI), using non-AR cohort without asthma history as the reference, were calculated to show the risk of GERD in the interaction analysis between AR and asthma. A Kaplan–Meier curve was used to estimate the probability of GERD, and the log-rank test was used to evaluate the differences between cohorts. All statistical operations were performed using the SAS 9.3 statistical package; all P-values were 2-sided, and P-values < 0.05 were considered significant.
